# Post-Translational Modification of Proteins Mediated by Nitro-Fatty Acids in Plants: Nitroalkylation

**DOI:** 10.3390/plants8040082

**Published:** 2019-03-29

**Authors:** Lorena Aranda-Caño, Beatriz Sánchez-Calvo, Juan C. Begara-Morales, Mounira Chaki, Capilla Mata-Pérez, María N. Padilla, Raquel Valderrama, Juan B. Barroso

**Affiliations:** Group of Biochemistry and Cell Signaling in Nitric Oxide, Department of Experimental Biology, Center for Advanced Studies in Olive Grove and Olive Oils, Faculty of Experimental Sciences, University Campus Las Lagunillas, University of Jaén, E-23071 Jaén, Spain; laranda@ujaen.es (L.A.-C.); sanchezcalvobeatriz@gmail.com (B.S.-C.); jbegara@ujaen.es (J.C.B.-M.); mounira@ujaen.es (M.C.); mmata@ujaen.es (C.M.-P.); npadilla@ujaen.es (M.N.P.); ravalde@ujaen.es (R.V.)

**Keywords:** nitro-fatty acids, nitroalkenes, nitroalkylation, electrophile, nucleophile, signaling mechanism, post-translational modification, reactive lipid species, nitro-lipid-protein adducts

## Abstract

Nitrate fatty acids (NO_2_-FAs) are considered reactive lipid species derived from the non-enzymatic oxidation of polyunsaturated fatty acids by nitric oxide (NO) and related species. Nitrate fatty acids are powerful biological electrophiles which can react with biological nucleophiles such as glutathione and certain protein–amino acid residues. The adduction of NO_2_-FAs to protein targets generates a reversible post-translational modification called nitroalkylation. In different animal and human systems, NO_2_-FAs, such as nitro-oleic acid (NO_2_-OA) and conjugated nitro-linoleic acid (NO_2_-cLA), have cytoprotective and anti-inflammatory influences in a broad spectrum of pathologies by modulating various intracellular pathways. However, little knowledge on these molecules in the plant kingdom exists. The presence of NO_2_-OA and NO_2_-cLA in olives and extra-virgin olive oil and nitro-linolenic acid (NO_2_-Ln) in *Arabidopsis thaliana* has recently been detected. Specifically, NO_2_-Ln acts as a signaling molecule during seed and plant progression and beneath abiotic stress events. It can also release NO and modulate the expression of genes associated with antioxidant responses. Nevertheless, the repercussions of nitroalkylation on plant proteins are still poorly known. In this review, we demonstrate the existence of endogenous nitroalkylation and its effect on the in vitro activity of the antioxidant protein ascorbate peroxidase.

## 1. Introduction

Reactive lipid species (RLS), or so-called lipid-derived electrophiles (LDEs), are caused by polyunsaturated fatty acids (PUFAs) peroxidation [1,2,3,4]. Reactive lipid species have been identified in sanguine fluid, plasma, urine, human tissues, and animal models using array techniques. Recently, they have also been detected in plant systems with the aid of mass spectrometry. A rise in RLS abundance under pathological and stress circumstances has been broadly reported [4,5,6,7,8,9,10].

Polyunsaturated fatty acids are targets of peroxidation due to their unsaturated double bonds [4,11]. The main mechanisms of PUFA peroxidation are non-enzymatic autocatalytic oxidation reactions [1,12], while enzymatic oxidation reactions involving three heme-containing metallo-enzyme families (lipoxygenases (LOXs), cyclooxigenases (COXs) [1,13], and cytochromes P450 (CYPs) [1]), as well as NADP^+^-dependent dehydrogenases [1,14] which can also occur. Non-enzymatic mechanisms include PUFA nitration triggered by reactive nitrogen species (RNS) such as nitric oxide (NO) and its derived molecules [1,15,16]. A preferential target for lipid peroxidation is arachidonic acid, whose oxidation yields several products. The non-enzymatic oxidation reactions of PUFAs yield aldehydes such as 4-hydroxynonenal (HNE) and malondialdehyde (MDA), as well as the J- and A-series of isoprostanes [4,17]. Prostaglandins (15-deoxy-Δ12,14-prostaglandin J_2_) and lipoxins are generated by enzymatic oxidation reactions catalyzed by COX and LOX, respectively [4,18,19]. The oxidation of arachidonic acid by NO-derived species yields 12-nitroarachidonic acid (12-NO_2_-AA) [4,20].

The addition of aldehyde, α-β-unsaturated carbonyl, epoxide or nitroalkene substituents to PUFAs during the peroxidation process causes the formation of lipid-derived species with electrophilic properties. From a chemical perspective, electrophilic molecules contain an electron-poor moiety, which makes them chemically reactive with nucleophiles (electron-rich molecules) [1]. Nucleophiles and electrophiles can be classified according to a hard/soft acid–base (HSAB) model [21]. Hard electrophiles, whose outer layer electrons are not readily excited, are difficult to polarize. Conversely, soft electrophiles have a more diffused electron distribution or partial positive charges due to the possession of electron-withdrawing substituents such as nitro groups. Nucleophiles can be characterized in a similar manner. Hard nucleophiles are highly electronegative and difficult to polarize, in contrast to soft nucleophiles, which have empty, low-lying electron orbitals. The softest biological nucleophiles, cysteine thiols, which integrate proteins, are also present in the antioxidant tripeptide glutathione (GSH). Primary and secondary amines of lysine, arginine, and histidine residues are regarded as hard nucleophiles [22]. The reactivity of nucleophiles does not only depend on the presence of hard and soft electrophiles in their vicinity, other factors such as their microenvironment (including hydrogen bonding reactions with neighboring amino residues) can influence nucleophile ionization too. For instance, as the reactivity of thiolate anions is higher than that of protonated thiols, the decrease in cysteine pKa induced by protein conformation increases its nucleophilicity [23,24]. As a general rule, hard electrophiles preferentially react with hard nucleophiles, while soft electrophiles interact with soft nucleophiles [1,25]. 

The importance of RLS resides in their electrophilic reactivity, which enables them to establish covalent adducts with GSH and nucleophilic amino acid residues of proteins such as cysteine, histidine, and lysine, generating post-translational modifications (PTMs) of proteins [4,26,27,28,29,30]. The endogenous occurrence of electrophilic fatty acids has been detected at low concentrations in plasma and animal tissues, whose biological significance is still little known [1,31]. Due to their innate reactivity, the rapid adduction process of RLS with susceptible GSH and nucleophilic residues of proteins may be functionally significant in relation to signaling responses [1,32]. However, it should be mentioned that an equilibrium between adducted and free forms exists in the milieu [1,33]. 

Pathological conditions promote the enzymatic and non-enzymatic generation of endogenous RLS. In these situations, an increase in the expression of oxidases and oxygenases and in the non-enzymatic production of reactive oxygen and nitrogen species (ROS and RNS), such as reduced oxygen species and oxides of nitrogen (NO, peroxynitrite (ONOO^−^), nitrogen dioxide (^·^NO_2_), nitronium cation (NO_2_^+^), takes place. All these species could react with PUFAs yielding RLS. Macrophage, eosinophil, and neutrophil cells in the immune system alter lipase activation, causing the scission of fatty acids from membranes. Thus, these disengaged fatty acids may be substrates for subsequent RLS formation [1,22,34]. The electrophilic nature of RLS induces the nucleophilic attack of proteins, leading to modifications in tertiary and quaternary structures, in catalytic activities, in charge and hydrophobicity, in subcellular localization, and in protein cross-linking. The main proteins susceptible to adduction perform metabolic functions such as cytoskeletal function, transcriptional regulation, host defense, ion and macromolecule transport, and enzyme catalysis. These proteins are involved in manifold physiological processes comprising resolution of inflammation, cell death, and induction of cellular antioxidants. In this respect, the anti-inflammatory and antioxidant responses stimulated by RLS adduction suggest the existence of an equilibrium between prompting events, electrophile production, protein adduction, and adaptive cellular responses. Therefore, RLS adduction allows organisms to cope with alterations generated under conditions of metabolic stress, inflammation, and modification in cells and tissues [1,4,35,36,37,38,39].

In plant systems, PUFA peroxidation caused by non-enzymatic or/and enzymatic (LOX-mediated) reactions generates some products with cytotoxic effects and others with protective anti-stress effects. The LOX pathway yields RLS related to plant defense responses to pathogen infections [40] and wounding [41], and in the regulation of hypersensitive programmed cell death [42] and senescence [43]. Non-enzymatic processes can generate both harmful products with damaging actions [44] and phytoprostanes, which have biological properties similar to those of jasmonic acid [45]. Recent knowledge has illustrated the formation of RLS that perform signaling roles and are implicated in antioxidant responses as a result of the oxidation of NO-derived molecules [9].

This review focuses on the study of reactive lipids species called nitroalkenes. Specifically, we will argue the biological properties of nitroalkenes both in animal and plant systems, as well as their signaling potential generated by a post-translational modification of proteins called nitroalkylation.

## 2. Nitro-Fatty Acids in Animals

The reactive lipids species resulting from the interaction of unsaturated fatty acids with NO and derived species, such as NO_2_ and ONOO^−^, are called nitro-fatty acids (NO_2_-FAs), nitrolipids or nitroalkenes [46]. 

**Figure 1:**
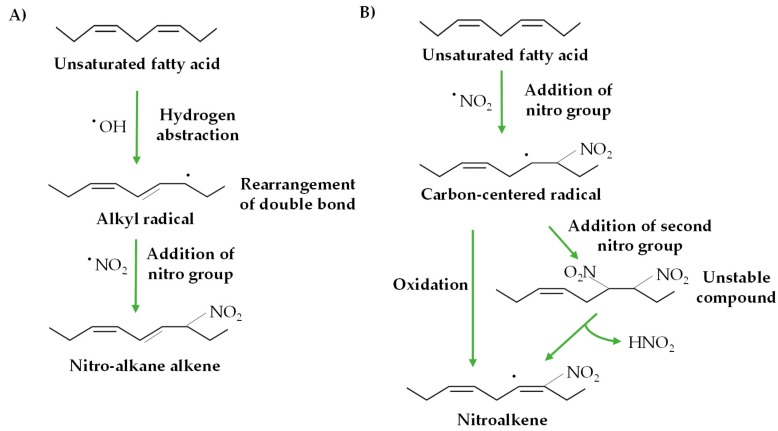
Possible mechanisms of nitrate fatty acid (NO_2_-FA) formation. (**A**) Alkyl radical generation through a radical hydrogen abstraction from a bis-allylic carbon followed by the insertion of NO_2_. (**B**) NO_2_-FA formation by the direct addition of NO_2_ and its oxidation (modified from Reference [49]).

Although the interaction between unsaturated fatty acids and RNS has been widely studied, two distinct mechanisms have been suggested to explain the in vivo nitration of fatty acids, a process which remains unknown [47]. One mechanism involves the generation of an alkyl radical through a radical hydrogen abstraction from a bis-allylic carbon followed by a double-bond rearrangement and the incorporation of a NO_2_ radical (Figure 1A) [48,49]. The other mechanism consists on the generation of a carbon-centered radical through the direct addition of NO_2_, which can be further oxidized either with or without a second insertion of NO_2_ in order to form the nitro-fatty acid. When the carbon-centered radical reacts with the second NO_2_, an unstable nitro-nitrite or dinitro compound appears which rapidly decomposes and releases nitrous acid (HNO_2_), yielding the nitro-fatty acid (Figure 1B) [49,50].

In recent years, important progress in the endogenous detection of NO_2_-FAs has been achieved in animal and human models. In animal systems, it is worth highlighting the detection of nitrated oleic (NO_2_-OA) and linoleic acid (NO_2_-LA) in the murine model of focal cardiac ischemia-reperfusion (I/R). The formation of these NO_2_-FAs was due to reoxygenation-induced tissue damage which generated acidification, hypoxia, as well as ROS and RNS [49,51]. It should be mentioned that other NO_2_-FAs were detected in an experimental rat model of ischemic preconditioning (IPC) [49,52]. High-resolution liquid chromatography mass spectrometry (LC-MS/MS) procedures have revealed a preferential nitration of conjugated linoleic acid (cLA) in animal systems. This fatty acid presents positional and geometric isomers of linoleic acid which have conjugated dienes in cis and trans configurations. These species have conjugated double bonds which are not separated by a methylene group [53]. Conjugated linoleic acid, which displays more reactivity with ^·^NO_2_ than bis-allylic fatty-acids, is the main in vivo endogenous nitration target [47,49]. The formation of nitrated cLA has been detected in activated macrophages under inflammatory conditions and in the gastric compartment following the ingest of cLA and NO_2_^−^ [47,49,54]. 

Advances in chromatography mass spectrometry techniques, in vitro nitration, and animal model studies have increased our understanding of the nitration of unsaturated fatty acids in humans. Dietary products such as oils and seeds are the principal sources of unsaturated fatty acids such as oleic acid (OA), conjugated linoleic (cLA), and linolenic (cLn) acids. Pomegranates are regarded as sources of cLn, while dairy products and meat are a source of cLA. Interestingly, cLA is absorbed at much higher levels than cLn [49,55]. Dietary products such as vegetables and herbs are sources of nitrate (NO_3_^−^) and nitrite (NO_2_^−^) [49,56,57]. These NO-derived species are necessary to generate nitrated PUFAs, as nitrite is a nitrating compound derived from nitrate. However, the low level of nitrite in basal metabolic conditions is increased through the conversion of nitrate by commensal bacteria in the gastrointestinal tract [58]. As with animal models, NO_2_-cLA is the principal nitroalkene generated in humans (Table 1) [47]. 

In addition to those mentioned above, other NO_2_-FAs, such as nitro-oleic acid (NO_2_-OA), nitro-linoleic acid (NO_2_-LA), conjugated nitro-linoleic acid (NO_2_-cLA), nitro-arachidonic acid (NO_2_-AA), and cholesteryl nitrolinoleate (NO_2_-CL) have been detected in vivo through quantitative analyses of blood and urine under both healthy and inflammatory conditions (Table 1) [59,60].

**Figure 2:**
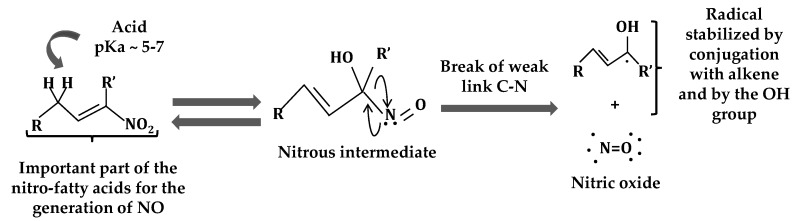
Mechanism of NO release through the modified Nef reaction. This mechanism consists of the generation of a nitrous intermediate which can homolyze in the aqueous medium to yield a carbon radical and nitric oxide (modified from Reference [62]).

**Figure 3:**
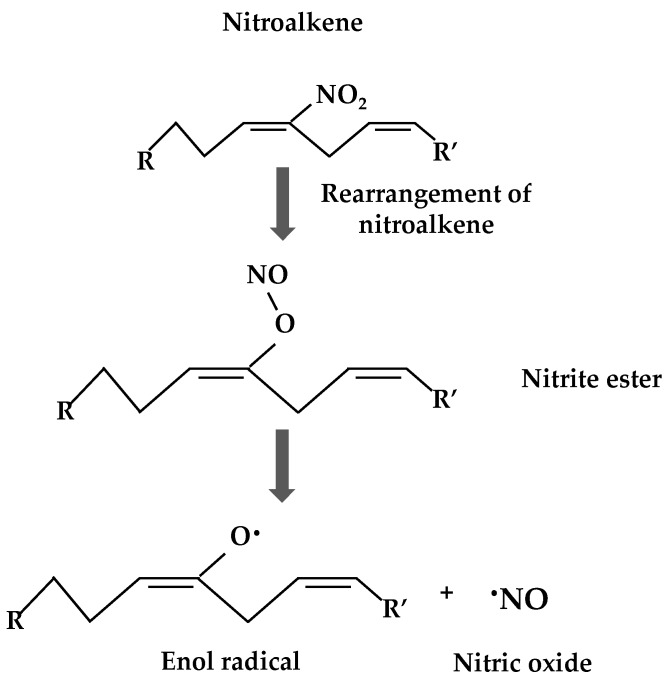
Release of nitric oxide from nitroalkenes through a rearrangement process. A nitrite ester is formed and homolyzed to yield NO and an enol radical (modified from Reference [15]).

Nitrate fatty acids are endowed with a specific chemical reactivity which facilitates cellular signaling events. In addition, these molecules have potent biological properties such as a NO-releasing capacity which was observed for the first time in aqueous milieu in animal systems [15,61,62,63]. Two possible NO-releasing mechanisms have been proffered. The first one consists of a modified Nef reaction which generates a nitrous intermediate with an especially weak C–N bond that quickly decays to yield NO and a radical stabilized by conjugation with alkene and the OH group (Figure 2) [15,46]. The second mechanism involves the rearrangement of the nitroalkene to a nitrite ester followed by a process of homolysis to form NO and an enol group (Figure 3) [46,64,65]. Another biological property of these compounds is their hydrophobic stability in cell membranes and lipoproteins, which act as endogenous NO_2_-FA reservoirs which can be supplied to other locations in the cell in order to act as signaling molecules [15]. An additional biological property of NO_2_-FAs is their capacity to mediate post-translational modifications through nitroalkylation, which will be discussed below [46,51,66,67,68].

Following the discovery of the presence of endogenous NO_2_-FAs and their biological properties in animal and human systems, their metabolism and distribution have been examined. In this regard, NO_2_-FAs have been shown to bind carrier proteins such as albumin, may be subjected to the normal lipid metabolism processes such as saturation and β-oxidation and can be esterified into complex lipids [22,49,69,70]. A recent study has shown that prostaglandin reductase leads to the reduction of NO_2_-FA to electrophilic nitroalkanes and that both alkenes and nitroalkanes are subjected to β-oxidation [71]. On the other hand, gastric digestion and inflammatory conditions lead to the formation of complex lipids containing NO_2_-FAs, as the formation of triglycerides (TGs) containing NO_2_-FAs has been detected in adipocytes and rat plasma following the in vitro acidic gastric digestion of TGs with NO_2_-OA supplementation [69]. Phospholipids containing NO_2_-FAs have also been uncovered in cardiac mitochondria and cardiomyoblasts from a diabetes mellitus animal model [72]. All these studies illustrate the presence of NO_2_-FAs and their metabolites in complex lipids. Lipase action can cause these NO_2_-FA-containing complex lipids to release electrophilic species. In addition, free electrophilic species may travel to remote tissues to regulate cell homeostasis and tissue signaling [49].

## 3. Nitro-Fatty Acids in Plants

Nitrate fatty acids have been widely regarded as novel mediators of cell signaling in animal organisms. However, the knowledge about them in the plant kingdom is limited. The constitutive presence of NO_2_-FAs in plant systems was initially characterized in extra-virgin olive oil (EVOO) (a basic component of the Mediterranean diet) in which oleic acid, followed by palmitic (PA), linoleic (LA), and linolenic (Ln) acids are present [73,74,75]. Given their properties mentioned above, the inherent occurrence of NO_2_-FAs in EVOO and olives was analyzed using mass spectrometry techniques. Different endogenous NO_2_-cLA isomers were detected in EVOO, while intrinsic NO_2_-OA-cysteine adducts (higher levels in the olive peel) were found in olives. These reports demonstrate that both EVOO and olives are sources and endogenous reservoirs of NO_2_-FAs, which could be responsible of the anti-inflammatory and anti-hypertensive properties of EVOO [10,70].

Additionally, the presence of NO_2_-FAs has been recently reported in both cell–suspension cultures (ACSC) and seedlings of the model plant *Arabidopsis thaliana*. Originally, the model plant’s lipid composition was analyzed, with a predominance of Ln, followed by LA and OA [10]. The biological occurrence of NO_2_-Ln (Table 1) was only detected in ACSC (0.28 pmol/g FW) and seedlings (3.84 pmol/g FW) [9,10], while a modulation in NO_2_-Ln levels was detected during plant growth. Seeds, 14-day-old seedlings and leaves from 30- and 45-day-old Arabidopsis plants were used. The higher NO_2_-Ln content (11.18 pmol/g FW) was quantified at the seeds stage, with a continuous decline observed in the final vegetative and reproductive stages of the life cycle (0.54 pmol/g FW) [9,10]. In addition, the potential of NO_2_-Ln to emit NO has been recently evidenced [9,76], and the high NO_2_-Ln content in seeds could provide an additional source of NO which could favor germination and the onset of vegetative development [9,77,78,79]. 

Mass spectrometry techniques were also used to analyze the profile of NO_2_-FAs in other plant species. In this sense, NO_2_-Ln was detected in rice (*Oryza sativa*) leaves (0.748 pmol/g FW). The same type of NO_2_-FA was identified in pea leaves (*Pisum sativum*) mitochondria (0.084 pmol/g FW) and peroxisomes (0.282 pmol/g FW) and roots (0.072 pmol/g FW). These analyses show the wide spread of NO_2_-FAs in plant organisms [10,76]. Furthermore, the levels of NO_2_-Ln detected in plants are similar to those found in animal systems [31], which reinforces their essential role as signaling contributors in plants [10,80].

On the other hand, the NO_2_-Ln abundance was also quantified in Arabidopsis under adverse environmental conditions such as mechanical wounding, salinity, low temperature, and heavy-metal stress. Under these stress situations, a meaningful rise in NO_2_-Ln content was monitored accompanied by an induction of genes associated with oxidative stress and oxygen-containing compound responses [9,80,81]. 

After demonstrating its relationship with plant development and plant adverse situations, a transcriptomic analysis by RNA-seq technology allowed us to analyze the signaling role played by NO_2_-Ln. Initially, ACSC treated with increasing concentrations of NO_2_-Ln (10 µM and 100 µM) showed this molecule’s clear signaling response in terms of plant physiology and dose-dependence responses [9] previously described in animal systems [47,82]. A set of overexpressed genes related to abiotic and oxidative stress responses were detected after treatment with NO_2_-Ln, while other genes implicated in biological procedures, such as biosynthesis of cellular metabolites, were downregulated, with a similar pattern being observed in seedlings. It is important to highlight the involvement of upregulated genes in protein folding as well as in responses to heat and H_2_O_2_ stress. Unexpectedly, around 40% of the genes which responded to NO_2_-Ln were involved in heat-shock responses (HSRs) [9]. In animal systems, the treatment with NO_2_-OA also activates a considerable number of genes related to HSRs, which reveals the presence of a conserved mechanism of response to NO_2_-FAs in both animal and plant systems [9,82].

Among the upregulated genes which responded to reactive oxygen species (ROS) is a gene encoding for cytosolic ascorbate peroxidase 2 (APX2), which is a relevant enzyme involved in defending plants against H_2_O_2_. Additionally, under abiotic stress situations such as high temperatures and light intensity, interactions between APX2 and the heat shock transcription factor (HSFA2) have been detected [10,83]. 

Although the participation of NO_2_-Ln in plant biology and responses to abiotic stress conditions has been previously described, the mechanisms involved in NO_2_-Ln’s defense responses to stress in plants are still little known. As with animal systems, the release of NO by NO_2_-Ln in aqueous medium, which could be a signaling mechanism, has been demonstrated in Arabidopsis cell cultures by various in vitro experimental techniques such as ozone chemiluminescence, 4,5-diaminofluorescein (DAF-2) spectrofluorometric probes, confocal laser scanning microscopy, and the oxyhemoglobin oxidation method. Ozone chemiluminescence showed that NO-releasing from NO_2_-FA was not propitious in acidic locations, since at neutral pH (7.4) the maximum releasing of NO was achieved. This finding may be of considerable importance inside the cells, as mitochondria, peroxisomes, and the cytosol have a basic or neutral pH [10,76]. In addition, when the leaves and roots of Arabidopsis seedlings were treated with NO_2_-Ln, green fluorescence arose as a consequence of the increase in NO content, thus demonstrating the in vivo capability of NO_2_-Ln to provide NO. In addition, the subsequent treatment of samples with the NO scavenger 2-(4-carboxyphenyl)-4,4,5,5-tetramethylimidazoline-1-oxyl-3-oxide (cPTIO) causes a decrease in fluorescence [9,84]. These results emphasize the important role of NO_2_-Ln as a NO reservoir, and thus, the indirect involvement of NO_2_-FAs in plant growth, in the response to (a)biotic stress processes and in a variety of NO-related post-translational modifications (NO-PTMs) [80,85,86,87].

## 4. Nitroalkylation

**Figure 4:**
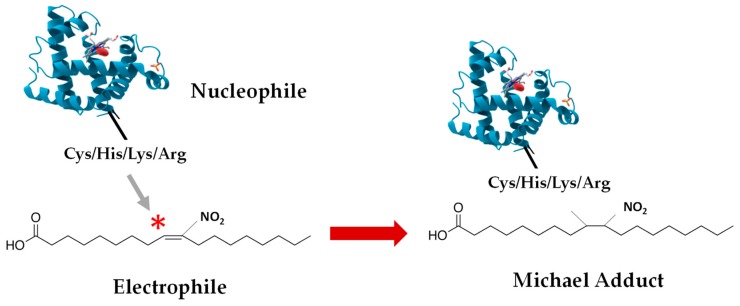
Nitroalkylation or formation of nitro-lipid-protein adducts. The attack of electrophilic nitro-fatty acids by nucleophilic protein residues leads to the establishment of a Michael adduct.

Nitro-fatty acids, which are potent electrophiles owing to the presence of electron-withdrawing nitro (-NO_2_) substituents in the beta carbon, mainly act via post-translational modifications. For this reason, they are able to react with nucleophiles like glutathione or target amino acid residues, which affects their protein structure and eventually their function and subcellular localization [67,88]. The nitroalkylation PTM involves the establishment of a nitro-lipid-protein adduct with the cession of a couple of electrons from the nucleophile to the electrophile (NO_2_-FA) to form a covalent bond, via a Michael adduction. This process generates lipoxidation adducts (Figure 4). Nitroalkylation provokes a chain of signaling phenomena that concludes with anti-inflammatory, anti-hypersensitive, anti-tumorigenic, cytoprotective, and antioxidant effects arbitrated by NO_2_-FAs [46,89]. 

Diverse studies have displayed the reversible character of nitroalkylation which enables it to act as a selective signaling pathway in stressful environments. Under these conditions, the rise in the ROS and RNS levels could affect the stability of nitroalkylation. Reactive oxygen and nitrogen species (ROS and RNS) can cause the oxidation of the bond between the sulfur residues and the α-carbon of the NO_2_-FAs (Michael adduct) resulting both in the generation of sulfoxides and derived species and the scission of the Michael adduct. This process results in the releasing of the nitroalkene which enables the protein to recover its initial state [22,66,81,88,90,91]. The reversible possibilities of nitroalkylation in biological processes are of considerable importance, as irreversible PTMs usually lead to permanent loss of function, and thus protein degradation [22,46,68]. Although the main nucleophiles which react with NO_2_-FA are cysteine thiols (Cys-SH), and not all are able to react with electrophiles, in this sense, the deprotonated cysteine thiolate (Cys-S^−^) is specifically the most prone to react [92,93]. Other nucleophiles are the amino substituents of lysine and arginine residues and the imidazole moiety of histidine [89].

### 4.1. Nitroalkylation in Animals

Nitrate fatty acids act as signaling mediators, since a scant amount of them act as powerful signal transduction cascade mediators that carry out changes in protein function through PTMs [1,66,68,94]. As mentioned above, processes such as digestion and inflammation lead to the genesis of NO_2_-FAs, predominantly NO_2_-cLA. In animal systems, NO_2_-FAs protect against a broad cluster of diseases such as atherosclerosis, restenosis, ischemia-reperfusion, renal injury, diabetes, metabolic syndrome, endotoxemia, and triple-negative breast cancer [95,96]. Their pluripotent cell signaling capacity enables NO_2_-FAs to modulate various intracellular pathways. In this line, the capacity of NO_2_-FAs to release NO via the Nef reaction generates low concentrations of NO which modulates cyclic monophosphate guanosine (cGMP)-dependent cell signaling activity. Nitrate fatty acids also control the generation of NO by regulating endothelial and inducible nitric oxide synthase (eNOS and iNOS) independently of cGMP mechanisms [34,62,76]. 

In addition, NO_2_-FAs can regulate the expression levels of differentiation-related, key inflammation, and cell proliferation genes [82,97,98,99,100,101]. Signaling via the Kelch-like ECH-associated protein 1 (Keap 1)-nuclear factor erythroid-derived 2-like 2 (Nrf2) pathway is a primary regulator of cellular responses to oxidative stress. The transcription factor Nrf2, which controls antioxidant protein expression, is located in the cytosol in its inactive form due to Keap1 activity which promotes Nrf2 ubiquitination and subsequent degradation by the ubiquitin–proteasome system. Keap 1 contains reactive cysteines (Cys 151, 273, and 288) which can be modified by oxidation or alkylation and used as redox state sensors. When electrophiles such as NO_2_-OA, NO_2_-LA, and NO_2_-AA are formed, the interaction between Nrf2 and Keap1 is interrupted. This facilitates the transfer of Nrf2 to the nucleus, where it will link to specific cis targets and activate the regulation of antioxidant response element (ARE) genes [1,55,97,102,103,104,105]. The NO_2_-FA-sensitive system involving heat-shock responses (HSRs) is a complex alliance of regulatory proteins and transcription factors which promotes cytoprotective and anti-inflammatory target gene expression [46]. Heat-shock proteins (HSPs) are chaperones whose expression is triggered by stress conditions, including heat, as well as by electrophilic and reactive species caused under inflammatory injury. Chaperones prevent the aggregation of denatured or oxidized proteins, collaborate in the transfer of these proteins to intracellular locations, and thus contribute to cellular redox homeostasis [106]. Nitro-oleic acid in human endothelial cells has been reported to activate HSF1 (Heat Shock Factor 1), the most important regulator of HSRs, followed by a remarkable induction of a large group of heat shock genes (Table 2) [82,102,107].

Nitro-fatty acid can also activate the peroxisome proliferator-activating receptor (PPAR), particularly PPARƴ, which is included in the family of nuclear hormone receptors. This receptor plays a marked role in the expression of transcription factors associated with lipid generation, lipid and glucose metabolism, macrophage differentiation, and immune responses. The PPARƴ regulatory domain is located in the C-terminal side which coincides with the ligand binding domain. The location of a cysteine at position 285 makes this hydrophobic region susceptible to nitroalkylation by NO_2_-FAs such as NO_2_-OA and NO_2_-LA (Table 2) [1,101,108,109,110].

Another example is the nuclear factor kappa betta (NF-kβ) involved in transcriptional regulation under inflammatory and immune processes. The nuclear factor kappa betta is a protein complex with two subunits (p50 and p65) [1,98,111,112]. Experimental studies have shown that NF-kβ is regulated by NO_2_-FAs at multiple levels including the inhibition of Toll-like receptor 4 (TLR4) by NO_2_-OA. Toll-like receptor 4 is a transmembrane protein which pertains to the pattern recognition receptor (PRR) family which is able to recognize bacterial lipopolysaccharide (LPS). Its activation triggers the intracellular NF-κB signaling pathway and inflammatory cytokine production which activate the innate immune system. Thus, the inhibition of TLR4 by NO_2_-FAs also triggers the inhibition of NF-kβ [101,113]. Another level of regulation is the inhibition of NF-kβ by nitroalkylation, specifically, the residue Cys38, placed in the DNA-binding domain of the p65 subunit, is susceptible to nitroalkylation [96,98]. The final level of regulation is the activation of PPAR by NO_2_-FAs which causes the trans-repression of inflammatory genes such as NF-kβ (Table 2) [101,114].

In animal systems, nitroalkylation is considered to be a decisive signaling resource in anti-inflammatory processes. Nitrate fatty acids modify the anti-inflammatory response at multiple levels including gene expression, protein translation (acting on transcription factors and lipid receptors), as well as cell function, as many inflammatory proteins contain numerous nucleophilic amino acid residues which can be nitroalkylation targets. Table 2 shows a summary list of NO_2_-FA protein targets in animal systems and how they are affected by nitroalkylation.

### 4.2. Nitroalkylation in Plants

Although the effects of nitroalkylation have been extensively studied in animal organisms, the impact of NO_2_-FA action in plants, which has not been fully explored, constitutes an emerging area of interesting research work. Probably, the signaling function of NO_2_-Ln is due to nitroalkylation processes. In this context, the endogenous presence of 37 proteins adducted with NO_2_-Ln in Arabidopsis cell cultures has been identified. However, cell cultures treated with 100 µM NO_2_-Ln showed an increase in the number of nitroalkylated proteins (342), belonging to different areas of cell metabolism, which included APX2 (unpublished results), whose encoding gene expression, according to the transcriptomic studies mentioned above, was induced [9]. 

Ascorbate peroxidase (APX2) is one of the primary antioxidant systems in plants. This enzyme belongs to the ascorbate–glutathione cycle, which detoxifies hydrogen peroxide and contains non-enzymatic antioxidants (ascorbate and glutathione) and enzymatic antioxidants such as monodehydroascorbate reductase (MDAR), glutathione reductase (GR), and dehydroascorbate reductase (DHAR), as well as the reductive coenzyme NADPH [127,128]. 

In this study, the APX recombinant protein from *Arabidospsis thaliana* was incubated with increasing concentrations of NO_2_-Ln (1 µM and 10 µM). The enzymatic activity was spectrophotometrically monitored [129]. Furthermore, the nitroalkylation targeted residues of the treated recombinant protein were detected and characterized using LC-MS/MS. Thus, the protein was digested by trypsin and desalted by C18 columns to obtain the peptide fraction which was analyzed using an Exactive Q mass spectrometer attached to a nano-flow liquid chromatograph (nanoLC) (Thermo Fisher Scientific). The LC-MS/MS spectrum deconvolution was carried out employing Proteome Discoverer version 1.4. bioinformatics software (Thermo Fisher Scientific). The Percolator node was used to filter the peptides at a 1% false discovery rate (FDR) at the peptide-spectrum matches (PSMs). 

In order to identify the position of the nitroalkylation-targeted nucleophilic residues, an in silico modeling was carried out using Raptor X bioinformatics software (http://raptorx.uchicago.edu/). The APX model was based on the structure of isoniazid (INH) bound to cytosolic soybean ascorbate peroxidase (PDB:2VCF) [130].

**Figure 5:**
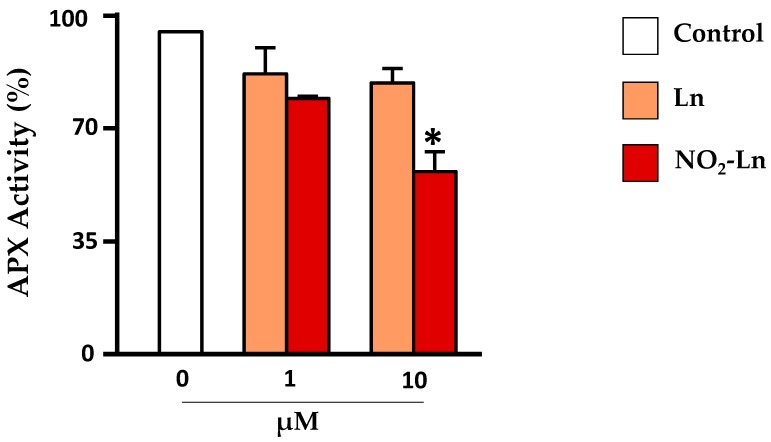
Modulation of the enzymatic activity of cytosolic recombinant APX following the treatment with increasing concentrations of NO_2_-Ln. The negative controls methanol (NO_2_-FA vehicle) and linolenic acid (non-nitrated fatty acid) were used. Vertical bars represent the mean ± standard deviation of at least three replicates. Statistically significant differences *p* < 0.05 (*) and *p* < 0.01 (**). (Ascorbate peroxidase: APX).

**Figure 6:**
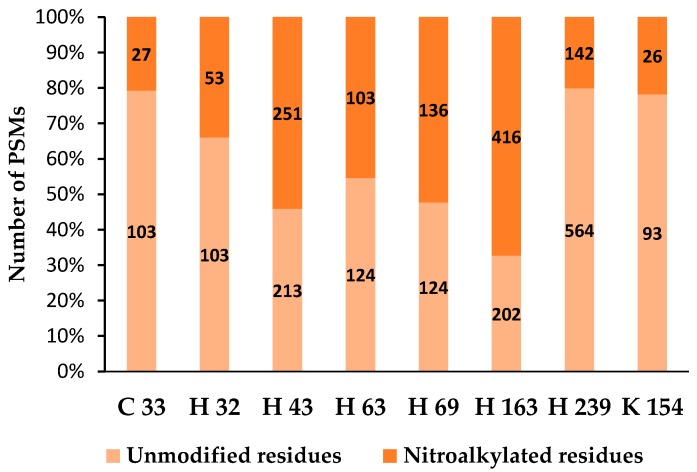
Detection of nitroalkylated residues in cytosolic recombinant APX by mass spectrometry (LC-MS/MS). The number on each column represents the number of PSMs of the unmodified residue related to the nitroalkylated residue. PSM: peptide-spectrum match.

**Figure 7:**
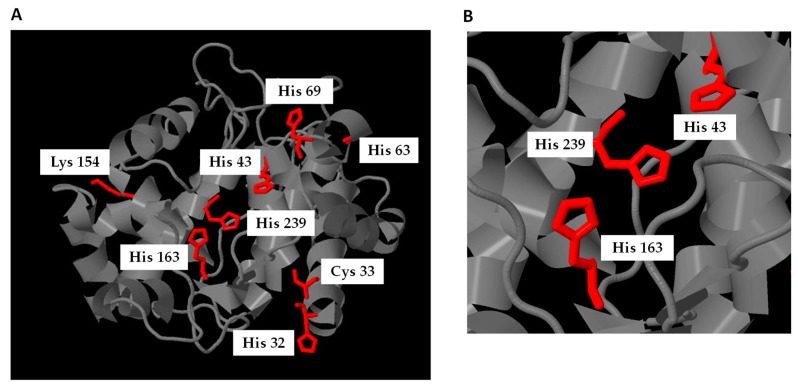
(**A**) In silico molecular model of cytosolic recombinant APX and localization of nitroalkylated residues. (**B**) Zoomed in illustration of the in silico molecular model where nitroalkylated histidines 43 and 163 located in the active site and in a metal-binding site, respectively, are highlighted.

The treatment of recombinant APX with NO_2_-Ln modulates its enzymatic activity, showing a significant decrease in the presence of 10 µM NO_2_^−^Ln (Figure 5). This decreased activity was associated with the post-translational modification caused by nitroalkylation, which was detected by mass spectrometry. Comparison of the spectra of control and NO_2_-Ln-treated samples displayed a rise in the mass of nucleophilic residues due to treatment with NO_2_-Ln. The electrophilic attack by NO_2_-Ln generated the nitroalkylation of the residues showed in Figure 6 and Figure 7A. with histidine 43 and histidine 163 being preferentially nitroalkylated. This could have functional implications (Figure 7B), as histidine 43 and histidine 163 are located at the active and metal-binding site, respectively. This fact suggests that the nitroalkylation of these residues blocks APX enzymatic activity, modulating protein function. 

**Figure 8:**
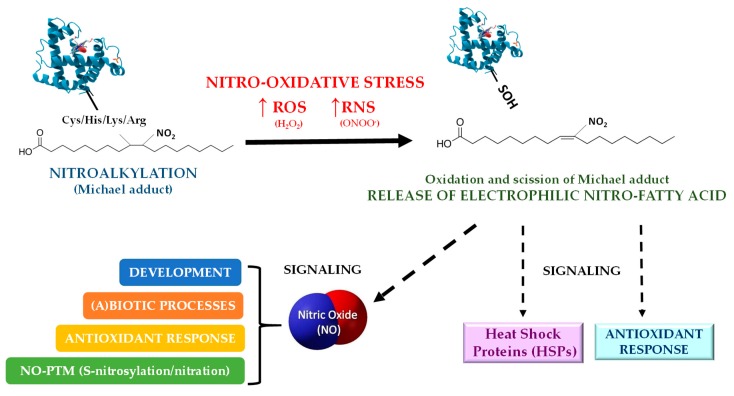
Model of the NO_2_-FA signaling mechanism by nitro-lipid-protein adduct in plants. Nitro oxidative conditions triggers the oxidation of the protein, the subsequent scission of the Michael adduct and the releasing of the NO_2_-FA. Free NO_2_-FAs display signaling actions by activating the chaperone network expression and several antioxidant systems. Moreover, NO_2_-FAs, which can also act as NO donors, are involved in NO signaling processes. ROS: reactive oxygen species; RNS: reactive nitrogen species; NO: nitric oxide.

Figure 8 explains the model of the nitro-lipid-protein adducts signaling mechanism in plants. Nitro-lipid-protein adducts stability can be affected by the accumulation of ROS and RNS, which could cause the oxidation of sulfhydryl substituents in proteins, and consequently the scission of the Michael adduct releasing NO_2_-Ln. As was previously mentioned, the nitroalkylation of APX by NO_2_-Ln generates function loss. Under nitro-oxidative conditions, the function of APX would be reactivated due to the reversibility of the nitroalkylation PTM. On the other hand, the levels of free NO_2_-FA increase, being able to stimulate the expression of heat shock proteins (HSPs) and certain antioxidant systems such as APX and methionine sulfoxide reductase B (MSRB). Another possibility is that NO_2_-FA could donate ^·^NO in the cellular aqueous environment which could act in a broad set of plant activities such as plant development, (a)biotic disorders, antioxidant responses, and NO-PTMs.

The ability of NO_2_-Ln to trigger pleiotropic signaling actions mainly depends on the nitroalkylation of regulatory proteins involved in plant biology and numerous types of (a)biotic-stress. Being a reversible post-translational modification, which can affect a large number of target amino acid residues (Cys, His, Lys, and Arg), together with the features outlined above, render nitroalkylation an important cell signaling mechanism mediated by NO_2_-FAs.

## 5. Conclusions and Future Perspectives

The potent electrophilic molecules NO_2_-FAs, whose electrophilicity triggers potential signaling mechanisms via nitroalkylation, were recently discovered in both animal and plant systems. This NO_2_-FA-mediated PTM can be considered a NO-PTM similar to *S*-nitrosylation, because NO_2_-FAs are RLS formed as a result of the oxidation of PUFA by NO-derived species. The importance of nitroalkylation resides in its reversibility and in the presence of a considerable amount of target amino acids residues that generate the formation of nitro-lipid-protein adducts, which enables this NO-PTM to trigger pleiotropic signaling actions. In animal systems, nitroalkylation is associated with signaling mechanisms in anti-inflammatory processes. However, in plant systems, this little-known NO-PTM constitutes an emerging area of research which should be developed through advances in mass spectrometry techniques. 

## Figures and Tables

**Table 1:** Principal nitro-fatty acids detected in animal and plant systems. The lines on the middle of the double bond indicate that the nitro group could be bounded in any of the adjacent carbons. Although double bonds can generate the corresponding cis- and trans-isomers, only the cis forms are shown.

Name	Formula	Chemical Structure
Nitro-oleic acid (9-, and 10-nitro-all-cis-octadecaenoic acid)	NO_2_-OA (18:1)	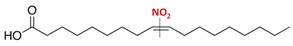
Nitro-linoleic acid (9-, 10-, 12-, and 13-nitro-all-cis octadecadienoic acid)	NO_2_-LA (18:2)	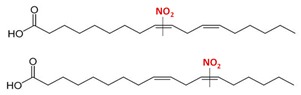
Nitro-linolenic acid (9-, 10-, 12-, 13-, 15- and 16-nitro-all-cis-octadecatrienoic acid)	NO_2_-Ln (18:3)	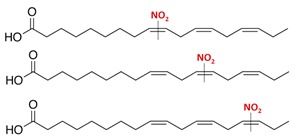
Nitro-arachidonic acid (5-, 6-, 8-, 9-, 11-, 12-, 14- and 15-nitro-all-cis-eicosatetraenoic acid)	NO_2_-AA (20:4)	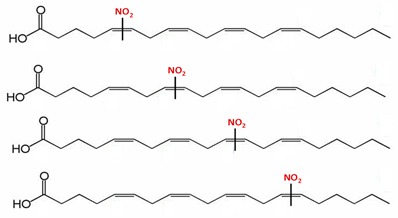

**Table 2:** NO_2_-FA protein targets in animal systems and their effects on protein function (modified from Reference [24]).

Nitro-Fatty Acid	Protein	Nucleophile Site	Effect	References
NO_2_-OA	GAPDH	Catalytic Cys, other Cys and His	Inhibition, increase in hydrophobicity and change in subcellular distribution	[66]
	Pro-MMP7 and Pro-MMP9	Zinc coordination Cys in the active site	Zinc release, autocatalytic cleavage of the pro-domain. MMP activation	[115]
	TRPV1 and TRPA1	Not detected	Activation of TRP channels	[116,117]
	AT1R	Not detected	Decrease in coupling with G-protein, inhibition of downstream signaling	[118]
	PknG	Iron coordination Cys in non-catalytic domain and His	Inhibition of kinase activity	[119]
	XOR	Pterindithiolene which coordinates molybdenum	Inhibition of electron transfer reactions at the molybdenum cofactor	[120]
	HSF1	Not detected	Activation of HSFA1 and subsequent robust induction of heat shock genes	[82,107]
NO_2_-LA	ANT1	Cys	Cardio-protection	[121]
NO_2_-cLA	HSA	Cys and non-covalent binding		[122]
NO_2_-AA	PGHS	Disruption of heme binding to the protein	Inhibition of PGHS-1 cyclooxygenase activity and both PGHS-1 and -2 peroxidase activity	[123]
	PKC	Probable covalent modification	Inhibitory effect on PKC activation	[124]
	NOX2	Inhibition of assembly	Inhibition of superoxide production	[125]
	PDI	Cys at active site	Inhibition of reductase and chaperone activities	[126]
NO_2_-OA and NO_2_-LA	NF-κB p65	DNA binding domain Cys	Inhibition of NF-κB DNA binding, abolition of pro-inflammatory responses	[98]
	PPARγ	Cys in ligand-binding domain	Agonist activation of PPARγ	[110]
NO_2_-OA, NO_2_-LA and NO_2_-AA	Keap 1	Cys	Stabilization of the complex with Nrf2, newly synthesized Nrf2 translocated to the nucleus	[97,103,104,105]

Abbreviations: Glyceraldehyde-3-phosphate dehydrogenase (GAPDH); Pro-matrix metalloproteinases, (Pro-MMP7 and Pro-MMP9); Transient receptor potential (TRPV1, TRPA1); Angiotensin II receptor (AT1R); Protein kinase G (PknG); Xanthine oxidoreductase (XOR); Heat Shock Factor 1 (HSF1); Adenine nucleotide translocase 1 (ANT1); Human serum albumin (HSA); Prostaglandin endoperoxide H synthase (PGHS); Protein kinase C (PKC); NADPH oxidase 2 (NOX2); Protein disulfide isomerase (PDI); Nuclear factor κB subunit p65 (NF-κB p65); Peroxisome proliferator-activated receptor (PPARγ); Kelch-like ECH-associating protein 1 (Keap 1).
